# Exploring Improved Supercapacitor Electrodes for Electrochemical Carbon Dioxide Capture

**DOI:** 10.1021/acselectrochem.6c00086

**Published:** 2026-04-24

**Authors:** Zhen Xu, Angus Pedersen, Shunsuke Shimizu, Takeharu Yoshii, Hirotomo Nishihara, Maria-Magdalena Titirici, Jesús Barrio, Alexander C. Forse

**Affiliations:** † Yusuf Hamied Department of Chemistry, University of Cambridge, Cambridge CB2 1EW, U.K.; # Department of Materials, University of Manchester, Manchester M13 9PL, U.K.; § Department of Chemical Engineering, Imperial College London, London SW7 2AZ, U.K.; ∥ Division 3.6, Electrochemical Energy Materials, Bundesanstalt für Materialprüfung und -forschung (BAM), 12203 Berlin, Germany; ⊥ Institute of Multidisciplinary Research for Advanced Materials, 13101 Tohoku University, Miyagi 980-8577, Japan; ¶ Advanced Institute for Materials Research (WPI-AIMR), 13101 Tohoku University, Miyagi 980-8577, Japan

**Keywords:** Porous carbon, supercapacitors, electrochemical CO_2_ capture

## Abstract

This study introduces a new porous carbon for electrochemical CO_2_ capture. Featuring both micro- and mesoporosity, it outperforms predominantly microporous YP80F (a commercial benchmark) by delivering faster CO_2_ adsorption and lower energy consumption. This highlights the importance of mesoporosity in designing improved supercapacitor electrodes for rapid, energy-efficient electrochemical CO_2_ capture.

## Graphical abstract

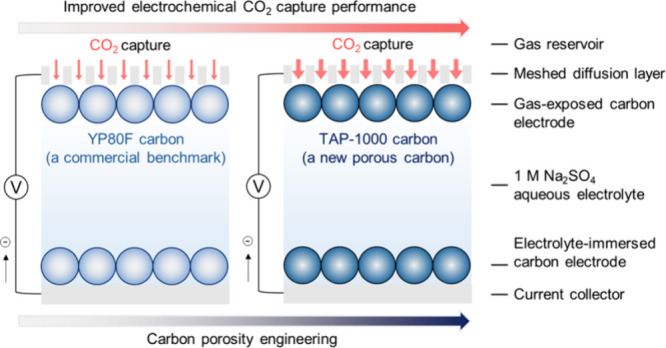

## Introduction

Anthropogenic CO_2_ is a major contributor to global climate change, necessitating the development of sustainable, energy-efficient CO_2_ capture technologies.^1^ Most conventional CO_2_ capture methods, such as amine scrubbing, require energy-intensive thermal regeneration processes, leading to high energy demands and degradation over repeated cycles.^2^ In contrast, electrochemical CO_2_ capture offers an energy-efficient alternative that enables reversible CO_2_ capture and release at room temperature by applying a voltage.^3,4^ Among various electrochemical CO_2_ capture technologies, supercapacitive swing adsorption (SSA) of CO_2_ is a promising candidate that operates by charging and discharging porous electrodes in a symmetric supercapacitor cell.^5^ During the negative charging of the gas-exposed electrode (Figure 1), CO_2_ is adsorbed by the supercapacitor cell, and during discharging, CO_2_ is released.^6^

**Figure 1:**
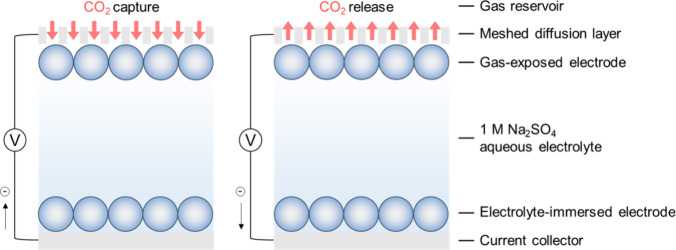
Schematic illustration of the electrochemical CO_2_ capture and release in a symmetric supercapacitor cell during the charge–discharge cycles.

Our previous studies revealed that SSA in the symmetric supercapacitor cell is intrinsically a kinetic effect that mainly occurs on shorter charging time scales, but the CO_2_ capture rate is limited by mass transport within the electrode under fast charging conditions.^7,8^ We therefore hypothesized that the mesoporosity of electrodes plays a critical role in enabling rapid CO_2_ capture by providing more accessible pathways for gas molecules and ions.^9^ Recently, Bilal *et al.* reported a series of advanced biomass-derived activated carbons with a combination of micro- and mesopores, which is also indicative of the importance of mesoporosity in improved SSA performance.^10^ However, a direct investigation into the role of mesoporosity development in enhancing CO_2_ capture performance is still lacking.

To address this gap, we present a new class of porous carbons synthesized from 2,4,6-triaminopyrimidine (TAP) and MgCl_2_·6H_2_O based on our previous work on carbon synthesis.^11,12^ This hard-templating method was developed for synthesizing carbon supports for supercapacitors and electrocatalysts with tunable porosity and high electrochemical active site utilization.^13^ Here, we used this strategy to prepare supercapacitor electrodes for electrochemical CO_2_ capture. During the synthesis, TAP was mixed with MgCl_2_·6H_2_O in a 1:8 weight ratio and subsequently carbonized at 900, 1000, and 1100 °C, yielding TAP-derived carbons with controlled pore structure after template removal. Because of the templating effect of the Mg^2+^ porogen, the TAP-derived carbons incorporate micropores and mesopores with controlled pore size distribution and pore volume. We focus on TAP-1000 (*i.e.*, TAP-derived carbon prepared at 1000 °C), which exhibits a high degree of mesoporosity while maintaining other structural characteristics (*e.g.*, carbon disorder) broadly comparable to the commercial benchmark YP80F. Based on this carefully designed materials platform of porous carbons, we establish a clear structure–property relationship linking mesopore structure to SSA kinetics using rate-dependent electrochemical analysis. In brief, this study presents a comparative analysis of the structural and electrochemical properties of TAP-1000 and YP80F, with a specific emphasis on their performance for fast, energy-efficient electrochemical CO_2_ capture.

## Results and Discussion

We first carried out structural characterizations to understand the differences between TAP-derived carbons and the commercial benchmark YP80F, as the structural properties of electrode materials are known to influence the SSA performance.^9,14^ N_2_ sorption measurements were employed to understand the pore structure of carbons (Figures 2a and S1). TAP-1000 shows a Brunauer–Emmett–Teller (BET) surface area of 2830 m^2^ g^–1^, higher than that of YP80F (*i.e.*, 2324 m^2^ g^–1^). The total pore volume of TAP-1000 is also larger, measured at 2.73 cm^3^ g^–1^, compared to 1.14 cm^3^ g^–1^ for YP80F (Figures 2b and S1 and Table S1). Both carbons exhibit a certain degree of microporosity, with pore widths centered near 0.9 nm (Figure 2b). The higher BET surface area and pore volume of TAP-1000 are therefore mainly attributed to the prominent new porosity spanning ∼1.5–10 nm with a dominant pore width around 2.4 nm, corresponding to mesoporosity. These parameters indicate that TAP-1000 may offer both greater surface area and faster transport pathways for ions and CO_2_.^15^ Raman spectroscopy (Figures 2c and S1) shows that TAP-1000 and YP80F exhibit similar degrees of structural disorder, with *I*_D_/*I*_G_ ratios of 1.17 and 1.16 (*i.e.*, the intensity ratio between the D and G peaks), respectively (Table S1).^16^ Temperature-programmed desorption (TPD) was employed to evaluate the chemical composition of the carbon bulk (Figure S2 and Tables S1 and S2).^17^ The results show that both carbons possess a small amount of oxygen (Figures 2d, S1, and S2 and Tables S1 and S2), while TAP-1000 possesses a nitrogen content of around 2.4 atom %.^18^ The remaining Mg content following synthesis in TAP-1000 is negligible (Table S1). This intermediate carbonization temperature (1000 °C) achieves an optimal balance between template-assisted pore development (via Mg species removal) and carbon framework stabilization, leading to maximized mesoporosity and accessible pore volume. In contrast, a lower temperature (900 °C) results in a higher heteroatom content and structural disorder of TAP-900, while a higher temperature (1100 °C) reduces the mesoporosity and surface area of TAP-1100 (Figure S1 and Table S1). Hence, compared to TAP-900 and TAP-1100, TAP-1000 exhibits the highest mesoporosity while retaining other structural characteristics broadly comparable to YP80F. These features make it the most suitable candidate for probing the effect of pore structure on electrochemical CO_2_ capture performance.

**Figure 2:**
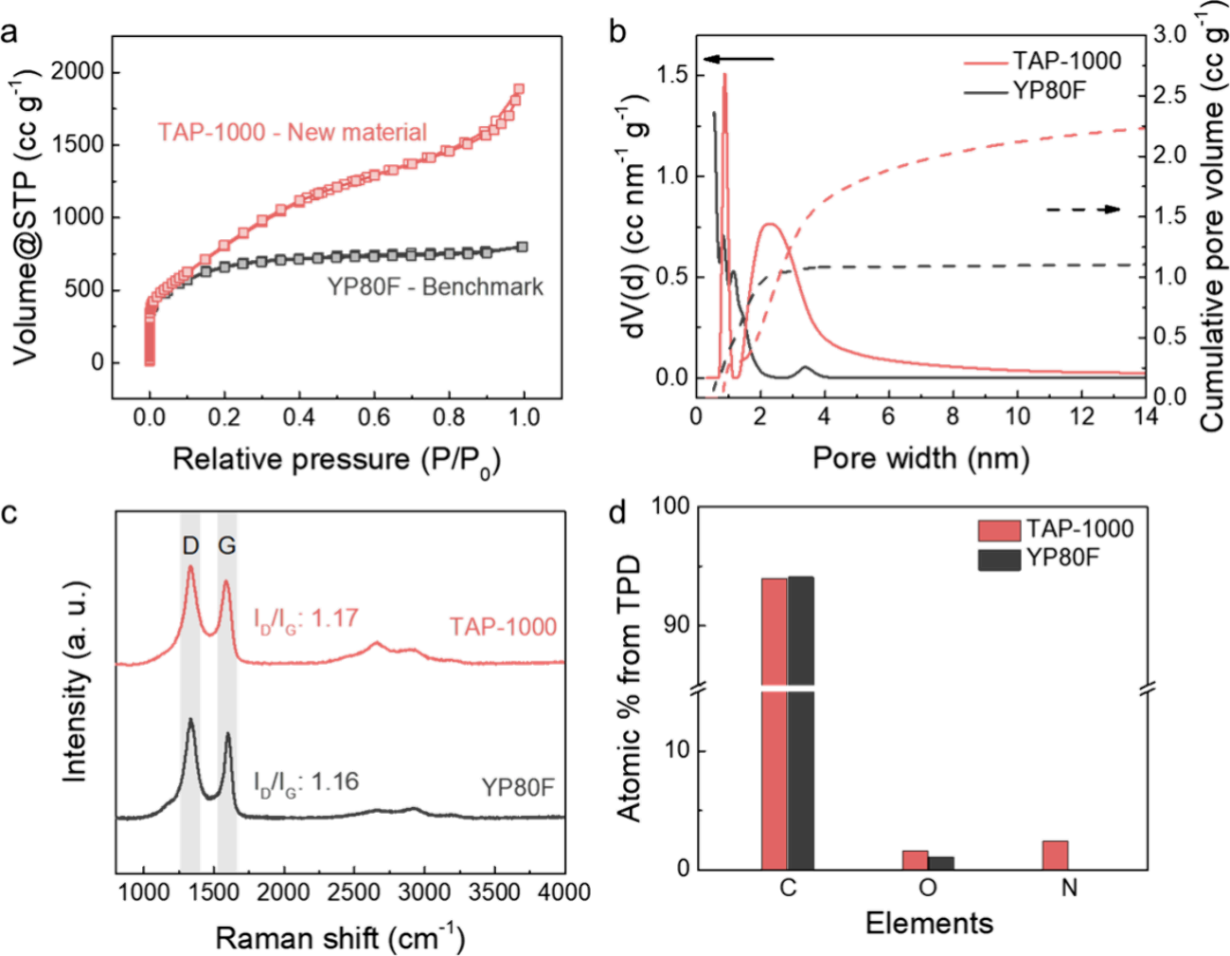
Characterization data for TAP-1000 and YP80F carbons. (a) N_2_ sorption isotherms at 77 K. (b) Pore size distribution and cumulative pore volume calculated using nonlocal density functional theory (NLDFT) and the slit pore model. (c) Raman spectra (532 nm). (d) Atomic percentage of elements in the bulk from TPD for TAP-1000 and YP80F.

After the assembly of symmetric supercapacitor cells with 1 M Na_2_SO_4_ aqueous electrolyte (Figures S3 and S4), electrochemical measurements were conducted. A capacitive cyclic voltammogram (CV) is observed for TAP-1000 and TAP-1100, consistent with the typical CV previously reported for YP80F,^19^ while TAP-900 has a resistive voltammogram even at 1 mV s^–1^ (Figure S5).^11^ TAP-1100 shows a smaller integral area of its CV curve than TAP-1000. These further identify TAP-1000 as the best candidate among TAP-derived carbons for comparison with YP80F.

The supercapacitor cell was then charged to a cell voltage of −0.8 V (termed “negative charging mode”, meaning that the CO_2_-gas-exposed working electrode was negatively charged) under a fixed current density (*i.e.*, 10, 70, or 150 mA g^–1^) (Figures 3 and S6). This voltage window was chosen to avoid the onset of water splitting.^20^ The differing time spans in Figure 3 arise from variations in current density, where lower currents lead to longer charge–discharge durations, and higher currents result in shorter cycles, despite the same number of cycles being shown. Additionally, the *y*-axis range of pressure is kept consistent (∼0.005 bar) in Figure 3, although the starting and ending pressure values differ between TAP-1000 and YP80F due to slight variations in the initial pressure after CO_2_ dosing in each experiment. Within each set of measurements, the pressure range is maintained across different current densities to enable a clear comparison of the pressure response as a function of the current density. As observed in previous studies, galvanostatic charge–discharge (GCD) profiles of the supercapacitor cell using YP80F electrodes reveal that CO_2_ capture occurs in negative charging, indicated by a measurable drop in CO_2_ pressure (Figure 3a). Upon discharging the cell back to 0 V, CO_2_ is released. The CO_2_ adsorption–desorption cycle is reproducible over multiple cycles. At the low current density of 10 mA g^–1^ (Figure 3a), the pressure response closely tracks the applied voltage, suggesting that there is sufficient time for CO_2_ sorption to proceed to completion. However, at the higher current densities of 70 and 150 mA g^–1^ (Figure 3b,c), a clear lag is observed between the voltage profile and the pressure response. Besides, the magnitude of the pressure change becomes diminished as the current is increased. These results indicate that at high current densities, CO_2_ capture becomes kinetically limited in the supercapacitor cell using YP80F, with insufficient time for CO_2_ adsorption and desorption to fully occur within each cycle. The cell using TAP-1000 electrodes shows some similarities with the YP80F cell (Figure 3d), with CO_2_ adsorption during negative charging and release during discharging. Unlike YP80F, the magnitude of pressure change with TAP-1000 becomes more pronounced as the current density is increased to 70 mA g^–1^, indicating an effective kinetic response for CO_2_ capture (Figure 3e). A slight decrease in pressure response is observed at 150 mA g^–1^, suggesting that while TAP-1000 performs well compared to YP80F at high rates, kinetic limits may still arise under very fast charging conditions.

**Figure 3:**
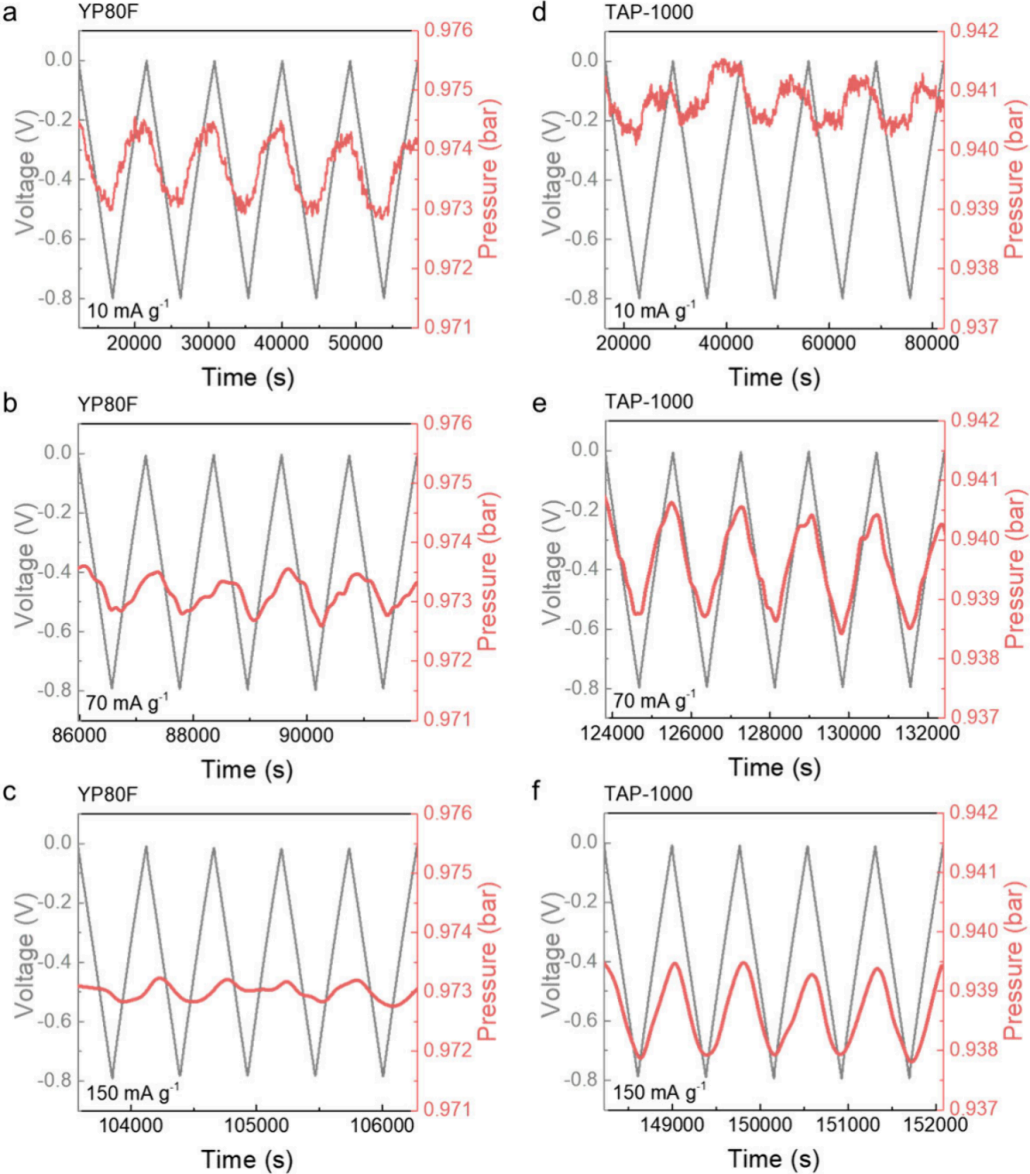
Electrochemical CO_2_ capture measurements. (a–c) GCD curves (gray) and corresponding pressure curves (red, smoothed every 100 s) of the YP80F//YP80F symmetric device (using 1 M Na_2_SO_4_(aq) as the electrolyte) for CO_2_ sorption in the negative charging modes at current densities of (a) 10 mA g^–1^, (b) 70 mA g^–1^, and (c) 150 mA g^–1^ (under CO_2_, without voltage hold after the charge or discharge process). (d–f) GCD curves (gray) and corresponding pressure curves (red, smoothed every 100 s) of the TAP-1000//TAP-1000 symmetric device (using 1 M Na_2_SO_4_(aq) as the electrolyte) for CO_2_ sorption in the negative charging modes at current densities of (d) 10 mA g^–1^, (e) 70 mA g^–1^, and (f) 150 mA g^–1^ (under CO_2_, without voltage hold after the charge or discharge process).

We then evaluated the electrochemical CO_2_ capture performance of YP80F and TAP-1000 across a broader range of current densities to investigate their rate-dependent CO_2_ capture behaviors (Figures 4 and S6). All the gravimetric performance metrics here were normalized by the mass of porous carbons in the working electrode. The cells with YP80F and TAP-1000 exhibit very similar IR drops at both low and high current densities (∼0.008 V at 30 mA g^–1^ and ∼0.07 V at 500 mA g^–1^) under identical conditions (Figure S6), suggesting that they have comparable internal resistance. However, the two carbons exhibit notable differences in gravimetric electrochemical capacitances. At a low current density of 5 mA g^–1^, YP80F delivers a capacitance of 119 F g^–1^, compared to 174 F g^–1^ for TAP-1000. Even at a high current density of 500 mA g^–1^, both carbons maintain relatively high specific capacitances, with YP80F at 98 F g^–1^ and TAP-1000 at 144 F g^–1^ (Figure 4a). The high capacitance retention for both carbons suggests fast charge storage kinetics in supercapacitors regardless of the differences in the pore structure between YP80F and TAP-1000. Yet TAP-1000 consistently delivers higher capacitance across all current densities, likely due to its larger BET surface area and pore volume.^21^

**Figure 4:**
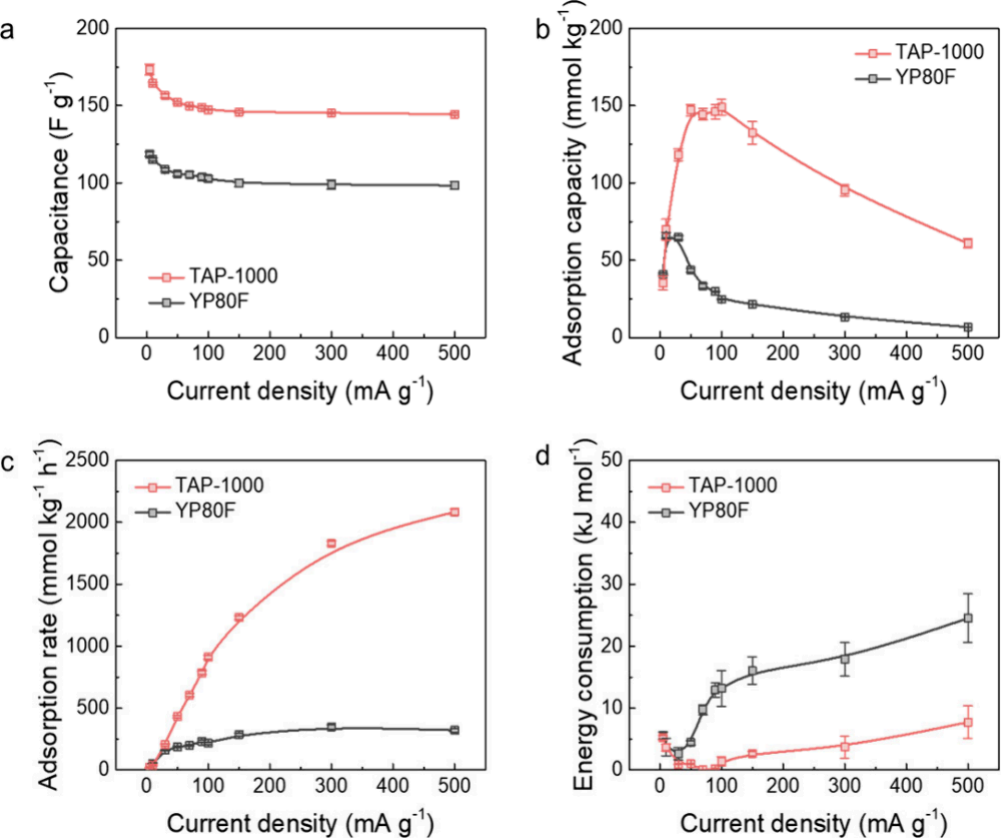
Electrochemical CO_2_ capture improvement. Characteristic dependences of (a) discharge capacitance, (b) electrochemical CO_2_ adsorption capacity, (c) CO_2_ adsorption rate, and (d) electrical energy consumption of the YP80F//YP80F and TAP-1000//TAP-1000 symmetric devices (using 1 M Na_2_SO_4_(aq) as the electrolyte at current densities from 5 to 500 mA g^–1^ in the negative charging mode without voltage hold under CO_2_.

In contrast, the gravimetric CO_2_ adsorption capacity (*i.e.*, the amount of CO_2_ captured per unit mass of electrode material with units of mmol_CO_2__ kg^–1^) shows significant differences for YP80F and TAP-1000 (Figure 4b). At 5 mA g^–1^, both carbons exhibit relatively modest CO_2_ adsorption capacities (*i.e.*, 41 mmol_CO_2__ kg^–1^ for YP80F and 35 mmol_CO_2__ kg^–1^ for TAP-1000). This region corresponds to “thermodynamic regimes” we have proposed previously, where competing CO_2_ capture and release processes at the two working and counter electrodes decrease overall CO_2_ capture uptake in symmetric supercapacitor cells.^7^ Specifically, the thermodynamic regime refers to conditions in which CO_2_ capture approaches its equilibrium capacity (typically at low current densities). Notably, the maximum CO_2_ adsorption capacities occur at different current densities for the two carbons. YP80F reaches its peak of 66 mmol_CO_2__ kg^–1^ at 30 mA g^–1^, while TAP-1000 achieves a higher maximum of 149 mmol_CO_2__ kg^–1^ at 100 mA g^–1^. When the current density further increases to 500 mA g^–1^, CO_2_ adsorption capacities decline for both carbons, but TAP-1000 clearly outperforms YP80F, with a CO_2_ adsorption capacity of 61 mmol_CO_2__ kg^–1^ compared to 7 mmol_CO_2__ kg^–1^ for YP80F. The differences in peak performance and the current density at which maximum CO_2_ uptake occurs highlight the superior kinetics of TAP-1000 for CO_2_ capture, which maintains high CO_2_ adsorption capacities even under rapid cycling. Performance reproducibility for repeat TAP-1000 and YP80F cells is presented in Figure S7.

We propose that the improved electrochemical CO_2_ uptake kinetics for TAP-1000 and the shift of the maximum adsorption capacity to higher current densities compared to YP80F are correlated with the greater mesoporosity of TAP-1000.^9^ To explore this hypothesis, we also evaluated electrochemical CO_2_ capture by TAP-1000 and TAP-1100. TAP-1100 presents a simple comparison, as it has heteroatom content and structural disorder similar to those of TAP-1000 but a lower surface area and pore volume due to the reduced mesoporosity mainly in the 1.5–3 nm and 4–10 nm ranges (Figures S1 and S2 and Tables S1 and S2). As a result, its CO_2_ adsorption capacities are lower nearly across all current densities, further highlighting the importance of mesoporosity (Figure S8). TAP-900 presents a more complex comparison, with higher nitrogen content than TAP-1000 along with a higher surface area and greater structural disorder (Figures S1 and S2 and Tables S1 and S2). It shows a slightly larger maximum CO_2_ uptake than TAP-1000 (Figure S9). However, the peak position of CO_2_ adsorption capacity of TAP-900 is shifted to a lower current density (TAP-900 peaks at 30 mA g^–1^ *vs* TAP-1000 at 100 mA g^–1^), and its adsorption performance declines rapidly at higher current densities, even with voltage holds, likely due to its poor electrical conductivity.^22^ This suggests that while the structural features of carbons like higher surface area and greater structural disorder may enhance thermodynamic capacities for CO_2_ capture, sufficient electrical conductivity is essential for maintaining CO_2_ capture performance at high current densities. According to our recent study,^23^ we also expect that carbon-pore-network tortuosity with respect to CO_2_ and CO_2_-derived ionic species may play an important role in the kinetic performance, and this will be measured in the future.

A more important performance metric for practical CO_2_ capture applications is the gravimetric CO_2_ adsorption rate (*i.e.*, the amount of CO_2_ captured per unit mass of electrode material per unit time with units of mmol_CO_2__ kg^–1^ h^–1^). For both YP80F and TAP-1000, the adsorption rate increases with increasing current density and eventually plateaus, reaching maximum values of 349 and 2083 mmol_CO_2__ kg^–1^ h^–1^, respectively (Figure 4c). When normalized by the density of the as-prepared electrodes, TAP-1000 maintains a substantial advantage on a volumetric basis (Figure S10). While the CO_2_ adsorption capacity per cycle decreases at higher current densities (Figure 4b), this is more than compensated by the shortened charge–discharge cycle times, resulting in enhanced CO_2_ adsorption rates. This contrasting trend between the adsorption capacity and rate emphasizes the kinetic advantages of rapid cycling of supercapacitors for electrochemical CO_2_ capture.

In the context of developing economically viable CO_2_ capture technologies, electrical energy consumption (*i.e.*, the electrical energy required to capture 1 mol of CO_2_ with units of kJ mol_CO_2__^–1^) is also a critical factor. Even under fast charging conditions (*e.g.*, 500 mA g^–1^), where the CO_2_ adsorption rate is maximized, we still observe very low energy consumption of <10 kJ mol_CO_2__^–1^ (Figure 4d). The low energy requirements underscore a key advantage of electrochemical CO_2_ capture using supercapacitors.^24,25^ However, it is worth noting that these measurements were conducted under pure CO_2_ conditions, which provides a standardized basis for directly comparing different carbons. Hence, the results do not directly represent performance under mixed-gas conditions, and a real CO_2_ separation would have a higher electrical energy consumption (Table S3). Using pure CO_2_ allows performance differences to be more directly attributed to structural features of YP80F and TAP-1000 without the added complexity of competitive gas transport and adsorption limitations in gas mixtures.^7^ A detailed CO_2_ selectivity study is given in our previous work.^9^ In brief, these results highlight the essential role of mesoporosity in enabling rapid, energy-efficient electrochemical CO_2_ capture, making it a critical design parameter for next-generation electrode materials.

## Conclusion

Summarizing, this study investigated the role of electrode pore structure, especially mesoporosity, in enhancing electrochemical CO_2_ capture by supercapacitors. A new class of porous carbons was synthesized from TAP, with TAP-1000 exhibiting a favorable combination of micro- and mesopores. Structural characterization confirmed that TAP-1000 has a higher BET surface area, larger pore volume, and greater degree of mesoporosity compared to the commercial benchmark activated carbon YP80F. Electrochemical CO_2_ capture measurements showed that TAP-1000 delivers higher capacitances, higher CO_2_ adsorption capacities, and much larger CO_2_ adsorption rates than YP80F, particularly under fast charging conditions. TAP-1000 achieved a maximum CO_2_ capture rate of 2083 mmol_CO_2__ kg^–1^ h^–1^ and maintained low electrical energy consumption (<10 kJ mol_CO_2__^–1^) even at a high current density of 500 mA g^–1^, which outperformed the commercial benchmark YP80F (349 mmol_CO_2__ kg^–1^ h^–1^ and 18 kJ mol_CO_2__^–1^ at 300 mA g^–1^). The results suggest that electrode mesoporosity is important in improving electrochemical CO_2_ capture performance, especially kinetic performance, enabling rapid, energy-efficient CO_2_ capture cycles. However, we cannot fully rule out a possible effect of the nitrogen content on electrochemical CO_2_ capture, requiring further investigation. Overall, this work highlights the importance of porosity engineering in designing high-performance supercapacitor electrodes for electrochemical CO_2_ capture. The measurements in this study were performed under static gas conditions, and ongoing work in our group is extending this study to flow-cell configurations to more closely evaluate the practical performance.

## Experimental Section

### Electrochemical CO_2_ Capture Measurements

Electrochemical gas adsorption experiments were performed using a custom-designed gas cell at 303 K (Figure S3).^26^ Electrochemical capacitors with a 1 M Na_2_SO_4_(aq) electrolyte were assembled within a coin cell with a meshed top case to allow gas access (SS316 CR2032, Cambridge Energy Solutions). During coin cell assembly, the gas-exposed electrode (*i.e.*, YP80F or TAP-1000, 12 mm diameter), the electrolyte-immersed electrode (*i.e.*, YP80F or TAP-1000), two 0.5 mm stainless steel spacers, one conical spring, two GF/A separators (Whatman, 20 mm diameter), and 200 μL of 1 M Na_2_SO_4_(aq) electrolyte were used. A potentiostat (VSP-3e or VMP-3e, Biologic) was used to conduct the electrochemical testing of gas cells, including the galvanostatic charge and discharge (GCD) measurement and cyclic voltammetry (CV). The gas adsorption or desorption was measured in a 30 °C incubator (SciQuip Incu-80S) by monitoring the gas reservoir pressure of the electrochemical gas cell with a pressure transducer (PX309-030A5 V, Omega).

## Supplementary Material



## Data Availability

The raw experimental data used in this study are available in the Cambridge Research Repository, Apollo, under accession code 10.17863/CAM.126379.

## Notes

Experimental details, characterization data, electrochemical measurements, and supplementary figures (PDF)
