# Supplementary material for: Exploring Improved Supercapacitor Electrodes for Electrochemical Carbon Dioxide Capture
Source: ACS Electrochem. 2026 Apr 24;2(5):1115–20. doi: 10.1021/acselectrochem.6c00086 (PMC13158909; doi:10.1021/acselectrochem.6c00086)
Supplement: Supplementary file 1 [file ec6c00086_si_001.pdf]

Supporting Information

Exploring improved supercapacitor electrodes for electrochemical carbon dioxide capture

Zhen Xu,[‡ab] Angus Pedersen,[‡cd] Shunsuke Shimizu,[e] Takeharu Yoshii,[e] Hirotomo Nishihara,[ef] Maria-Magdalena Titirici,*[cf] Jesús Barrio,*[c] and Alexander C. Forse*[a]

[a] Yusuf Hamied Department of Chemistry, University of Cambridge, Cambridge CB2 1EW, UK

[b] Department of Materials, The University of Manchester, Manchester M13 9PL, UK.

[c] Department of Chemical Engineering, Imperial College London, London, SW7 2AZ, UK

[d] Division 3.6, Electrochemical Energy Materials, Bundesanstalt für Materialprüfung und -forschung (BAM), Berlin 12203, Germany.

[e] Institute of Multidisciplinary Research for Advanced Materials, Tohoku University, Miyagi 980-8577, Japan

[f] Advanced Institute for Materials Research (WPI-AIMR), Tohoku University, Miyagi 980-8577, Japan

‡ Equally contributed.

* Corresponding authors.

Email: m.titirici@imperial.ac.uk, j.barrio-hermida@imperial.ac.uk, acf50@cam.ac.uk

Contents:

Materials and Methods

Figure S1-S10

Table S1-S3

Materials and Methods

TAP carbon synthesis

2,4,6-triaminopyrimidine (TAP) (97% Sigma Aldrich) and magnesium chloride hexahydrate (99% Sigma Aldrich) were ground with a pestle and mortar in a weight ratio of 1:8, respectively, and the mixture was pyrolysed in a ceramic crucible (filled 1/3rd with material) at 900-1100 °C for 1 h (at set temperature) under N_2 atmosphere (>99.998%, BOC) with 300 mL min^{-1} flow-rate and 5 °C min^{-1} heating rate. After heating the furnace was then allowed to cool naturally. The materials were collected, ground to fine powder, and washed with 2 M HCl (prepared by dilution of fuming 37% HCl, Merck) overnight to remove remaining $MgCl_2$ and MgO species. After the washing process, the powders were filtered, rinsed with 500 mL distilled water, dried at 80 °C under vacuum overnight, and labelled as TAP-X (X represents the annealing temperature) (Figure S1).

Electrode fabrication

Electrodes were prepared using porous carbons and polytetrafluoroethylene (PTFE) binder, with a weight ratio of 95:5 for YP80F and 90:10 for TAP-X. YP80F was purchased from Kuraray. For the electrode fabrication, around 200 mg of carbon was dispersed in 5 mL of absolute ethanol (Sigma Aldrich) and combined with a PTFE dispersion (60 wt% dispersion in H_2O, Sigma Aldrich), followed by stirring for roughly an hour to attain a dough-like consistency after ethanol evaporation. The mixture was subsequently rolled on a glass sheet with a roller (0.25 mm thickness for YP80F and 0.5 mm thickness for TAP-1000 due to different carbon densities) to create a free-standing electrode. This electrode was transferred onto aluminium foil and dried in a vacuum oven at 95 °C overnight. Circular electrodes with a diameter of 0.5 inches (around 12 mm) were cut out to achieve an approximate mass of 10-15 mg for CO_2 capture testing purposes.

Gas physisorption analysis

The pore structures of carbon materials were tested using N_2 sorption isotherms (Micromeritics 3Flex system and Anton Parr Autosorb iQ-XR) at 77 K. Before the testing, samples were degassed at 200 °C under vacuum for 16 h. The Brunauer–Emmett–Teller (BET) specific surface area was obtained by taking the adsorption isotherm in the relative pressure range of <0.3 and the best region for linear fit of 10 points ($R^2 > 0.995$) as determined by the Rouquerol method, and pore size distributions were obtained using the non-local density functional theory (NLDFT) and slit pore model.[1]

Temperature programmed desorption

Temperature programmed desorption (TPD) measurements were conducted using a recently developed system designed for precise analysis of H, O, and N contained in carbon materials.[2] The system consists of a reaction unit and a gas analysis unit. The reaction unit included a high-frequency induction heating system (EASYHEAT 8310LI, Ambrell) and a high-purity graphite sample holder (PYROGRAPH, Toyo Tanso, 99.9999%) placed inside a quartz chamber with water cooling. Holder temperature was monitored via a radiation thermometer. The gas analysis unit consisted of a quadrupole mass spectrometer (QMS, MPH-100M, Inficon Co., Ltd.), a calibrated gas reservoir, and a stainless-steel high-vacuum line evacuated using a turbo molecular pump. Prior to TPD runs, the empty graphite holder was pretreated at 2100 °C under high vacuum (2×10^{-6} Pa) for 1 hour. A 1-2 mg sample, weighed with a high-precision balance (Sartorius SE2), was then loaded into the holder. The

system was evacuated and heated from room temperature to 2100 °C at a rate of 10 °C min^{-1}. Evolved gases (H_2, H_2O, CO, CO_2, NH_3, HCN, and N_2) were quantified using the calibrated QMS, with calibration curves established from known gas concentrations supplied by the reservoir. Chemical species with the same m/z value, such as CO and N_2 (m/z = 28), were distinguished and quantitatively analysed by referring to their respective fragment ratios, based on previously reported studies.[2]

Inductively coupled plasma mass spectrometry analysis

For inductively coupled plasma mass spectrometry (ICP-MS) analysis, the samples were digested in 10 mL of aqua regia (25 v/v% HNO_3, 70%, Certified AR, Eur.Ph., Fisher Chemical; 75 v/v% HCl, 37%, Certified AR, Eur.Ph., Fisher Chemical) using a MARS 6 microwave digestion system operated at 1,500 W for 15 min at 215 °C. After digestion, the solutions were diluted and analysed using an Agilent 7900 ICP-MS (Agilent Technologies) with external calibration standards of 0, 10, 50, 100, 200, and 500 ppb.

Raman spectroscopy

Raman spectra were acquired using a Renishaw inVia confocal Raman microscope with a 532 nm laser excitation source. Spectra were collected with a laser power of 2.5 mW (0.5% of the 500 mW maximum output), an acquisition time of 10 s, and two accumulations per scan. For each sample, three to four spectra were recorded and averaged. For four-peak analysis, all spectra were baseline-subtracted, normalised to the maximum intensity, and deconvoluted using a four-peak Lorentzian fitting model. Fit parameters included peak positions, full widths at half maximum (FWHM), and peak areas. The I_D/I_G ratio was calculated using the fitted D and G peak intensities.

Electrochemical CO_2 capture measurements

Electrochemical gas adsorption experiments were performed using a custom-designed gas cell at 303 K.[3] Electrochemical capacitors with a 1 M Na_2SO_4 (aq) electrolyte were assembled within a coin cell with a meshed top case to allow gas access (SS316 CR2032, Cambridge Energy Solution) (Figure S3). During coin cell assembly, the gas-exposed electrode (*i.e.,* YP80F and TAP-1000, Diameter: 12 mm), the electrolyte-immersed electrode (*i.e.,* YP80F and TAP-1000), two 0.5 mm stainless steel spacers, one conical spring, two GF/A separators (Whatman, Diameter: 20 mm) and 200 µL of 1 M Na_2SO_4 (aq) electrolyte were used. After assembly, all components including electrodes in the meshed coin cell were firmly stacked together with a fixed total cell thickness of 3.2 mm. For symmetric supercapacitors, two identical carbon films with the same mass were used. After that, the meshed coin cell was inserted in the gas cell with the mesh side facing the gas reservoir, followed by the filling of the gas reservoir with pure CO_2 (99.80% purity, BOC), N_2 (99.998% purity, BOC) or O_2 (99.5% purity, BOC). For air-to-CO_2 exchange in the gas reservoir, a gas manifold was employed (Figure S4). To prevent electrolyte evaporation, the cell was subjected to a static vacuum. Subsequently, the valve closest to the cell was shut, and the gas manifold was dosed with CO_2 at around 1.3 bar. The decreased pressure in the gas cell aids the mixture of the gas reservoir with CO_2 from the manifold upon opening the cell valve. Then the cell valve was closed, and the manifold returned to dynamic vacuum. This dosing process was iterated 4 more times to establish an approximately pure CO_2 headspace.

A potentiostat (VSP-3e and VMP-3e, Biologic) was used to conduct the electrochemical testing of gas cells including the galvanostatic charge and discharge measurement (GCD) and cyclic voltammetry (CV). The gas adsorption or desorption was measured in a 30 °C incubator (SciQuip Incu-80S) by monitoring the gas reservoir pressure of the electrochemical gas cell with a pressure transducer (PX309-030A5V, Omega). The noise of the pressure transducer is at the level of 0.1 mbar, and the signal-to-noise ratio is over 5, which indicates a reasonable sensitivity of the pressure sensor. We also averaged the pressure data every 100 seconds to further decrease the effect of random pressure noise. In addition, we validated the pressure transducer using the two additional pressure sensors (MKS PDR2000 Dual Capacitance Manometer) on the gas manifold with accuracy at the level of 0.01 mbar (Figure S4), ensuring high measurement accuracy and reliability.

Considering the challenges associated with equilibration time in static gas methods (*i.e.,* electrochemical gas capture measurements without gas flow) and the slower gas diffusion rates, all gas cells were pre-cycled under 1 mV s^{-1} for 20 cycles (~ 8 hours) during which time CO_2 continued to equilibrate with the cell. The 1-hour rest before the regular GCD measurement was associated with a horizontal pressure baseline, which indicates the established equilibrium of the whole system after pre-cycling (Figure S6). All the electrochemical CO_2 capture measurements were repeated using at least two independent cells to confirm the reproducibility.

Calculations

The specific discharge capacitance values of the working electrodes were calculated from GCD (galvanostatic charge-discharge) measurements according to Equation S1, as follows:[4]

(1) $$C_{electrode} = 2\frac{I\Delta t}{m\Delta U}$$

where $C_{electrode}$ (F g^{-1}) refers to the specific discharge capacitance of the working electrodes. I (A) is the constant charge/discharge current, ΔU (V) is the change range of the cell voltage (for the hybrid capacitor, it is the change range of the potential), m (g) is the mass of active material (*i.e.,* porous carbon) loaded on the working electrodes, and Δt (s) is the discharge time under galvanostatic discharging. The cell voltage of the whole device is:

(2) $$\Delta U = U_{max} - IR_{drop} - U_{min}$$

where U_{max} and U_{min} are the maximum and minimum voltage applied, respectively.

The input/output energy values of the full device were obtained using Equation S3, as follows [3]:

(3) $$E_{in/out} = \frac{I}{m}\int_{t_1}^{t_2} U(t)dt + \frac{U}{m}\int_{t_3}^{t_4} I(t)dt$$

where $E_{in/out}$ (kJ kg^{-1}) is the input/output energy normalized by the active mass of the working electrode. I (A) is the constant charge/discharge current, m (g) is the active mass of the working electrode, and U (V) is the constant voltage/potential. U(t) (V) is the voltage/potential that changes with time under galvanostatic discharging or charging, t_1 (s) is the start time of galvanostatic charge/discharge processes, and t_2 (s) is the end time. I(t) (A) is the current that changes with time during voltage/potential hold, t_3 (s) is the start time of voltage/potential, and t_4 (s) is the end time.

The Coulombic efficiency (CE, %) was calculated to evaluate the reversibility of the stored charges using

equation S4, as follows:

(4) $$CE = \frac{\int_{t_3}^{t_4} I(t)dt}{\int_{t_1}^{t_2} I(t)dt} \times 100\%$$

where I(t) (A) is the current that changes with time. t_1 and t_2 (s) are the start time and end time of charge processes, respectively. In addition, t_3 and t_4 (s) are the start time and end time of discharge processes, respectively.

The specific gravimetric CO_2 adsorption capacity (C_{CO2}, $mmol_{CO2}$ kg^{-1}) was calculated by taking the difference between the maximum and minimum peaks of the gas amount (Δn, mol) in the reservoir and normalized by the mass of porous carbons in the working electrode. An average of two minimum points is used to avoid the error from the irreversible pressure changes when calculating adsorption capacity. This was converted from the pressure transducer data (smoothed every 100 seconds for all experiments) using the ideal gas law:[3]

(5) $$n = \frac{pV}{RT}$$

(6) $$\Delta n = \frac{n_{max_1} + n_{max_2}}{2} - n_{min}$$

(7) $$C_{CO_2} = \frac{\Delta n}{m} \times 10^6$$

where p, V and T are the pressure (Pa, 10^{-5} bar), volume (m^3, 10^6 mL) and temperature (K) respectively, and n is the gas amount (mol). R is the ideal gas constant (8.31451 m^3 Pa mol^{-1} K^{-1}), and m (g) is the active mass of the working electrode. In a completed cycle of CO_2 adsorption and desorption, n_{max1} and n_{max2} represent the two maximum peaks of the gas amount, and n_{min} represents the minimum peak of the gas amount. The overall adsorption capacity was taken as the mean of adsorption capacities for 6 cycles, and the error was calculated using a 95% confidence interval with the Student's t-test of performance from cycle to cycle at the same current density.

The specific volumetric CO_2 adsorption capacity ($C_{CO2\text{-}vol}$, $mmol_{CO2}$ L^{-1}) was calculated by the density of working electrode (ρ, kg L^{-1}).

(8) $$C_{CO_2-vol} = C_{CO_2} \times \rho$$

The volume of the gas reservoir (V_1, mL) in the gas cell was calculated during the process of dosing CO_2 into the cell, based on pressure measurements of the added gas using Boyle's Law ($P_1V_1 = P_2V_2$). For each gas cell, the volume in a section of pipe between two valves was known from a prior calibration (*A* mL) (Figure S4). First, the gas between two valves was removed using a vacuum, and the gas amount in the main gas reservoir was proportional to P_1V_1, where P_1 (bar) was read from the pressure sensor (Figure S6). After that, we allowed gas to enter the evacuated portion of the known volume (*A* mL). During this process, the total amount of gas remained the same (therefore, $P_1V_1 = P_2V_2$) but the pressure decreased to P_2 (bar) (Figure S6). As V_2 (mL) was equal to the sum of V_1 (mL) and *A* (mL), the volume of the reservoir (V_1, mL) was calculated using Equation S8:

(9) $$V_1 = \frac{P_2A}{P_1 - P_2}$$

This step was also used as the leaking test to make sure there was no leaking of the gas cell. If the gas cell leaks, the pressure will not be maintained at the low pressure of P_2 (bar) < 1 bar (Figure S6).

The specific electrical energy consumption (E, kJ mol_{CO2}^{-1}) were calculated using Equation S9, as follows:[5-6]

(10) $E = \frac{E_{in} - E_{out}}{C_{CO_2}} \times 10^3$

Figures and Tables

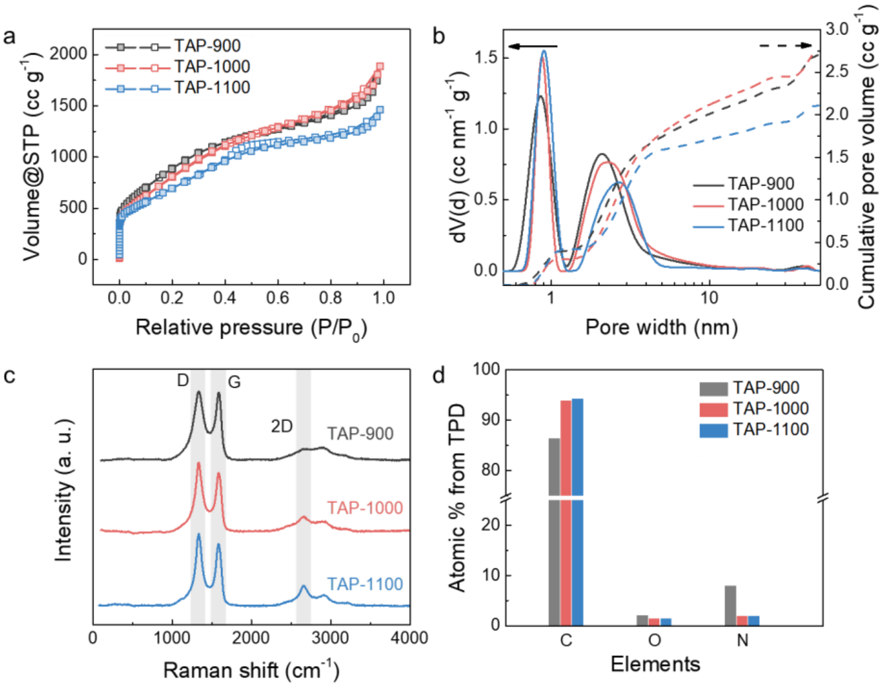

Figure S1. Structural analysis of porous carbons. (**a**) N_2 sorption isotherms at 77 K, (**b**) Pore size distribution and cumulative pore volume calculated using the non-local density functional theory (**NLDFT**) and slit pore model, (**c**) Raman spectra (532 nm), (**d**) Atomic percentage of elements in the bulk from TPD, of TAP-900, TAP-1000, and TAP-1100. Note: TAP-1000 was selected for comparison with YP80F as it exhibits the highest meso-porosity among the TAP-derived carbons, while maintaining other structural characteristics broadly similar to YP80F, making it the most appropriate candidate for isolating the effect of pore architecture on CO_2 capture performance.

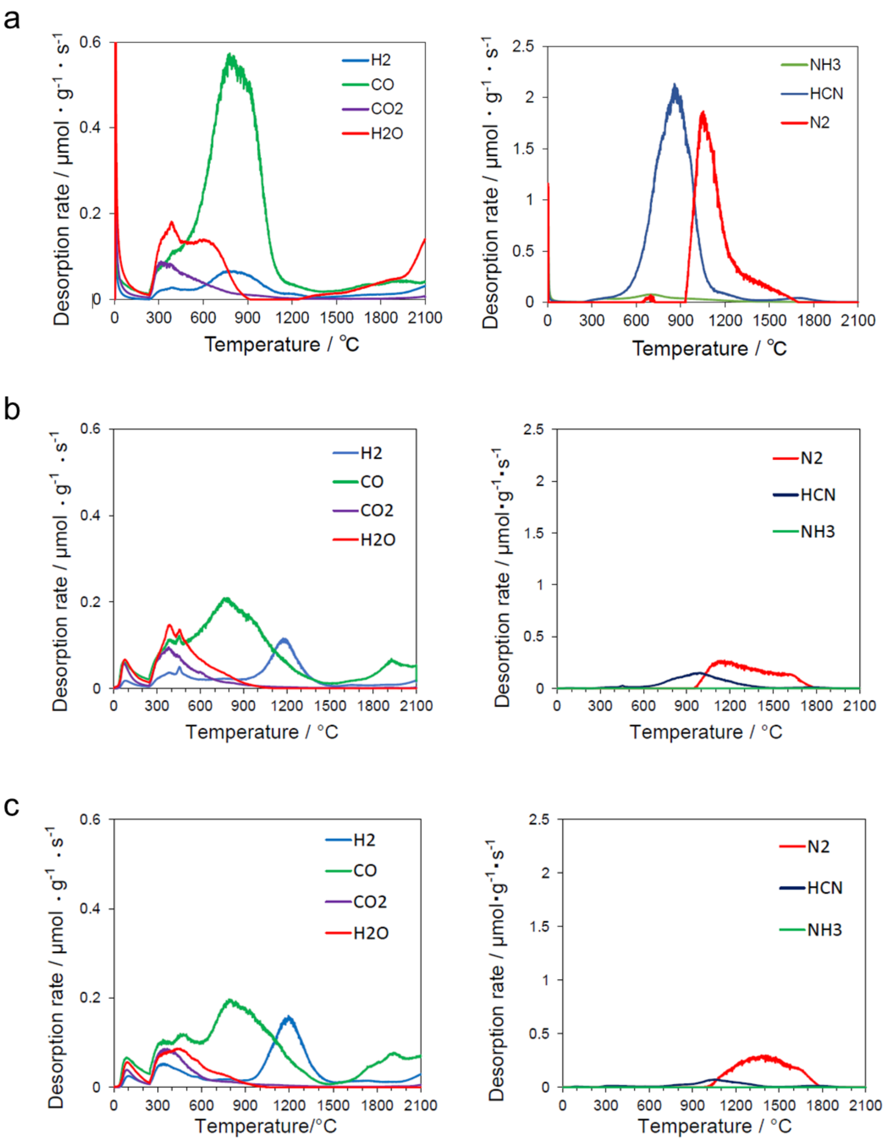

Figure S2. TPD measurements of porous carbons. TPD profiles of (**a**) TAP-900, (**b**) TAP-1000, and (**c**) TAP-1100 for the thermal desorption products of H_2, CO, CO_2, H_2O, N_2, HCN, NH_3.

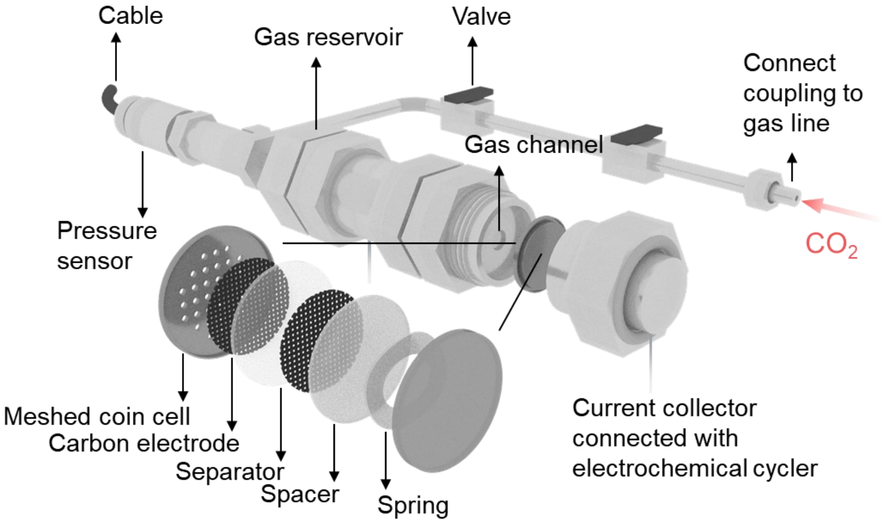

Figure S3. Schematic of the custom-made gas cell setup that houses a meshed coin cell for electrochemical CO_2 capture measurements at 303 K.

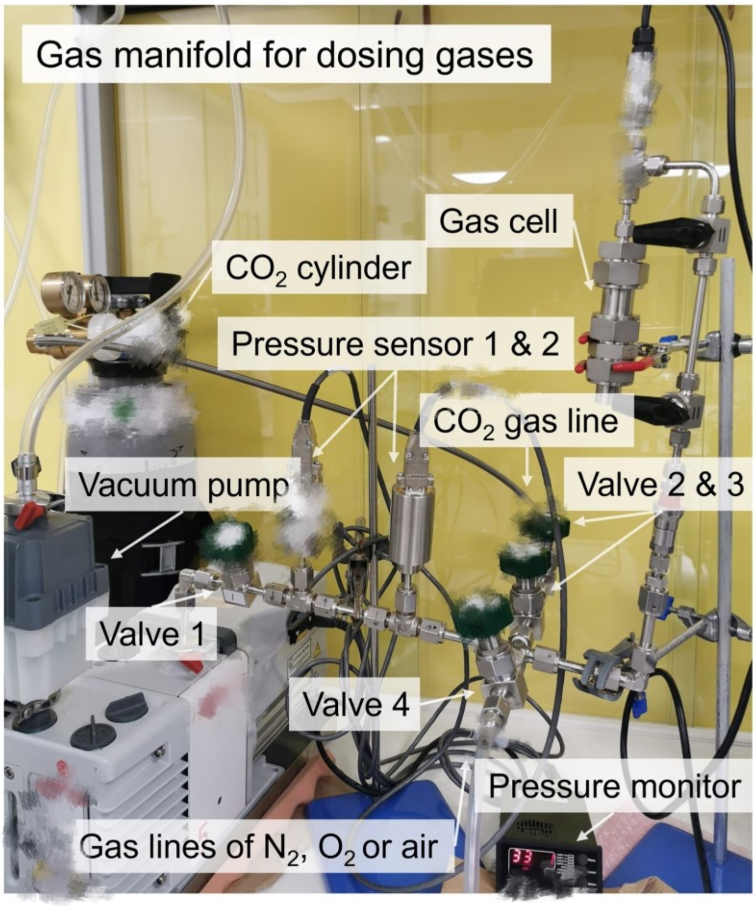

Figure S4. The mechanistic models and the setup of the gas manifold for dosing gases. A photo of the gas manifold for dosing gases into the electrochemical gas cell, where Valve 1 was used to control the connection between vacuum pump and gas cell, Valves 2 & 3 were used to control the connection between CO_2 cylinder and gas cell, and Valve 4 was used to control the connection between other gas cylinders and gas cell.

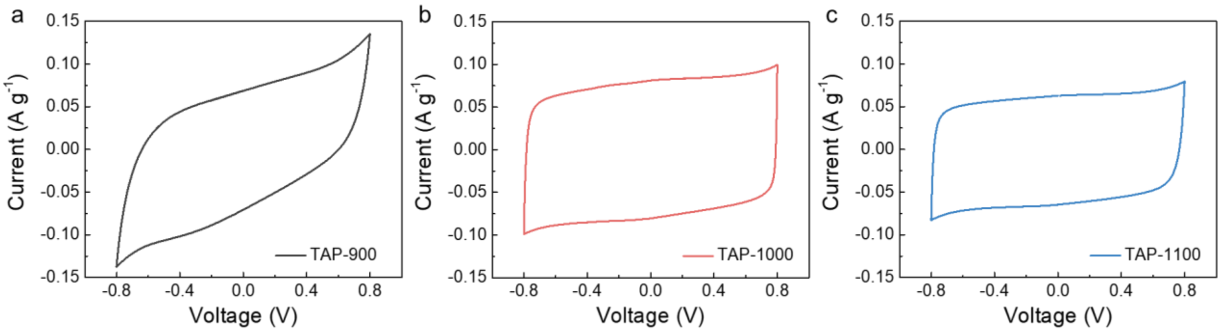

Figure S5. CV curves of the symmetric devices using (**a**) TAP-900, (**b**) TAP-1000 and (**c)** TAP-1100 (using 1 M Na_2SO_4 (aq) as the electrolyte) (under CO_2, at the scan rate of 1 mV s^{-1}). Note: The rectangular CV profile of TAP-1000 exhibits typical electrochemical double-layer capacitance (EDLC) behaviour, indicating good electrical conductivity and confirming its high capacitance among the TAP-derived carbons. In contrast, TAP-900 has a notably resistive voltammogram, which may relate to its lower electrical conductivity,[7] while TAP-1100 has an obviously smaller integral area of voltammogram, suggesting its lower charge storage capacity.

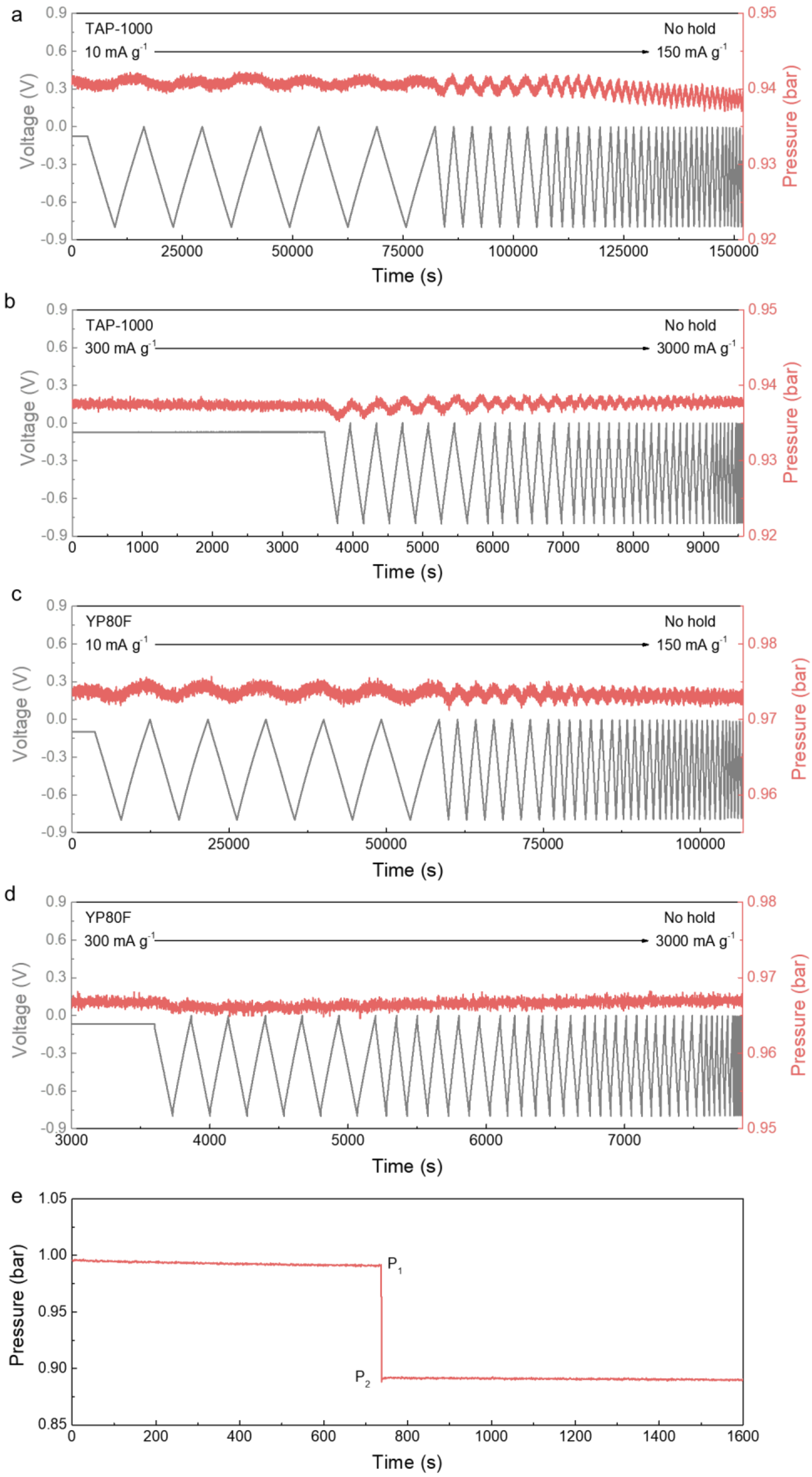

Figure S6. Overall GCD curves (grey) and corresponding pressure curves (red) of the TAP-1000//TAP-1000 symmetric device (using 1 M Na_2SO_4 (aq) as the electrolyte) at different current densities (**a**) from 10 to 150 mA g⁻

^{1} and (**b**) from 300 to 3000 mA g^{-1} in the negative charging mode, all without voltage hold. Overall GCD curves (grey) and corresponding pressure curves (red) of the YP80F//YP80F symmetric device (using 1 M Na_2SO_4 (aq) as the electrolyte) at different current densities (**c**) from 10 to 150 mA g^{-1} and (**d**) from 300 to 3000 mA g^{-1} in the negative charging mode, all without voltage hold. (**e**) Pressure curves (red) of the TAP-1000//TAP-1000 symmetric device before and after gas was allowed to enter the evacuated portion between the two valves of the electrochemical gas cell. (All under CO_2)

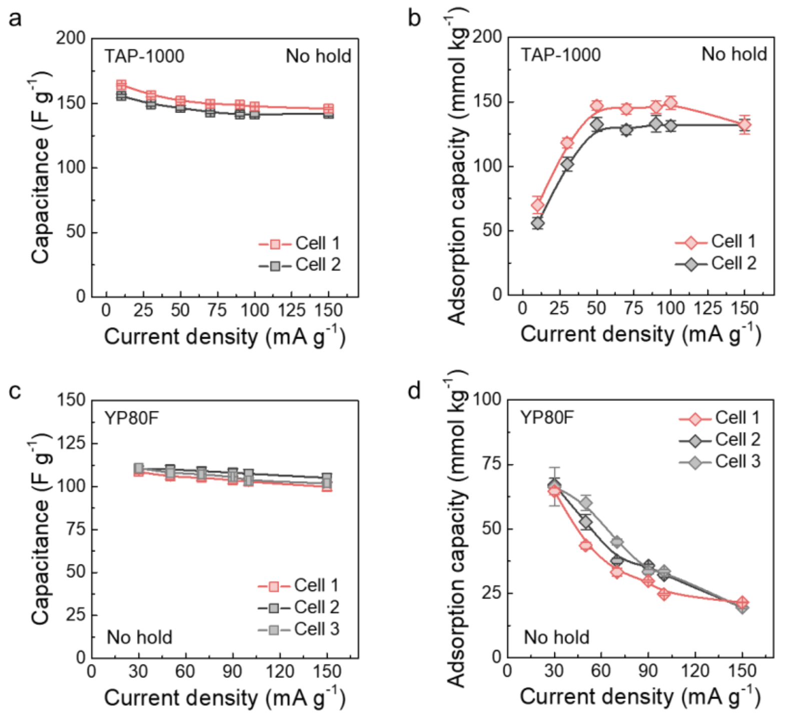

Figure S7. Tests of the reproducibility of electrochemical CO_2 measurements. Characteristic dependence of (**a**) the discharge capacitance (under CO_2), (**b**) electrochemical CO_2 adsorption capacity, of TAP-1000//TAP-1000 symmetric devices (using 1 M Na_2SO_4 (aq) as the electrolyte) (under CO_2, at the current densities from 10 to 150 mA g^{-1} in the negative charging mode, without voltage hold). Characteristic dependence of (**c**) the discharge capacitance (under CO_2), (**d**) electrochemical CO_2 adsorption capacity, of YP80F//YP80F symmetric devices (using 1 M Na_2SO_4 (aq) as the electrolyte) (under CO_2, at the current densities from 30 to 150 mA g^{-1} in the negative charging mode, without voltage hold). Cell 1 is the cell presented in the main text.

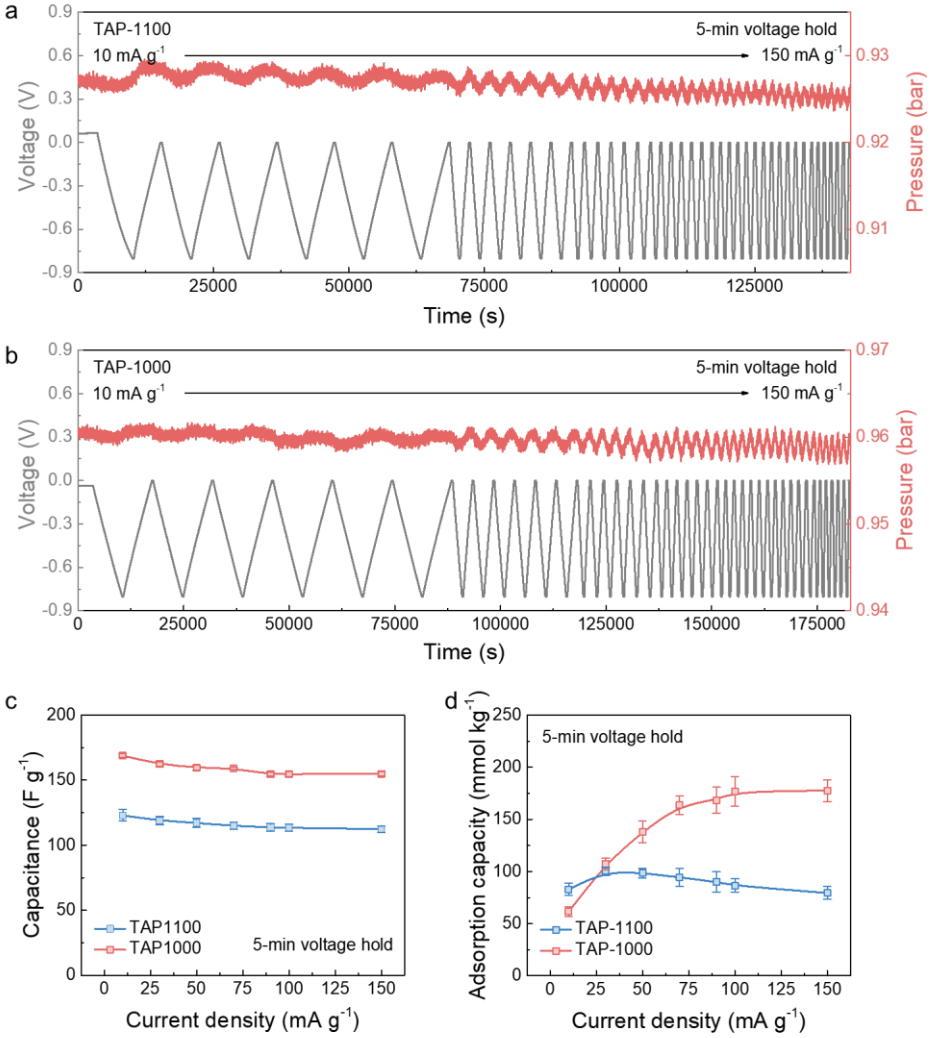

Figure S8. Overall GCD curves (grey) and corresponding pressure curves (red) of (**a**) the TAP-1100//TAP-1100 symmetric device (using 1 M Na_2SO_4 (aq) as the electrolyte) and (**b**) the TAP-1000//TAP-1000 symmetric device (using 1 M Na_2SO_4 (aq) as the electrolyte) at different current densities from 10 to 150 mA g^{-1} in the negative charging mode, with a 5-min voltage hold between charging and discharging (under CO_2). Characteristic dependence of (**c**) the discharge capacitance (under CO_2), and (**d**) electrochemical CO_2 adsorption capacity of the TAP-1100//TAP-1100 and TAP-1000//TAP-1000 symmetric devices (under CO_2). Materials characterisation shows that TAP-1100 has a similar heteroatom content and structural disorder to TAP-1000. However, its surface area and pore volume are lower, primarily due to reduced meso-porosity between ~1.5 and 3 nm. Consistent with these features, TAP-1100 exhibits lower capacitances than TAP-1000 at all current densities (129 *vs.* 169 F g^{-1} at 10 mA g^{-1}; 112 *vs.* 155 F g^{-1} at 150 mA g^{-1}), although both show comparable capacitance retention with increasing current density. Similarly, TAP-1100 displays lower CO_2 adsorption capacities at nearly all current densities (except 10 mA g^{-1}), with a maximum uptake of 100 $mmol_{CO2}$ kg^{-1} at 30 mA g^{-1} compared to 177 $mmol_{CO2}$ kg^{-1} for TAP-1000 at 100 mA g^{-1}. These observations indicate that pore structure strongly influences both the thermodynamic and kinetic performance of CO_2 capture, whereas for charge storage it mainly affects the thermodynamic capacity. This highlights the critical role of meso-porosity in enabling efficient electrochemical CO_2 capture.

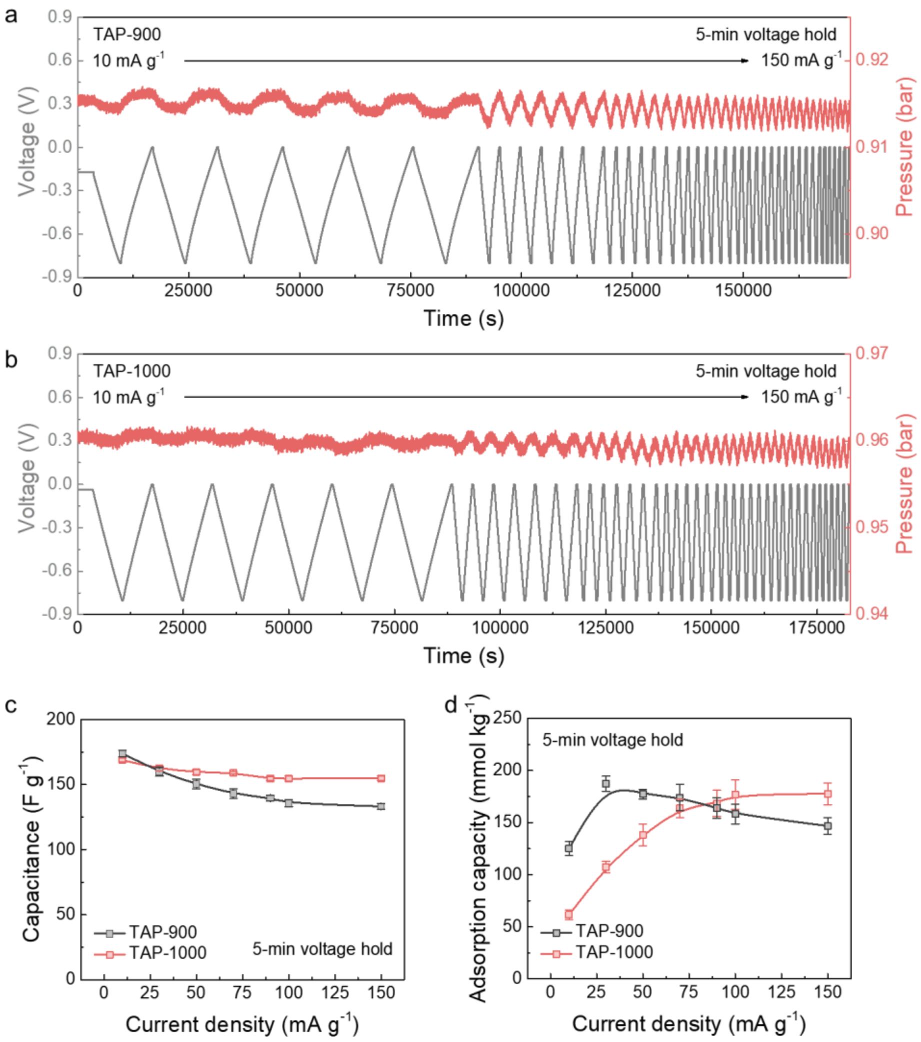

Figure S9. Overall GCD curves (grey) and corresponding pressure curves (red) of (**a**) the TAP-900//TAP-900 symmetric device (using 1 M Na_2SO_4 (aq) as the electrolyte) and (**b**) the TAP-1000//TAP-1000 symmetric device (using 1 M Na_2SO_4 (aq) as the electrolyte) at different current densities from 10 to 150 mA g^{-1} in the negative charging mode, with a 5-min voltage hold between charging and discharging (under CO_2). Characteristic dependence of (**c**) the discharge capacitance (under CO_2), and (**d**) electrochemical CO_2 adsorption capacity of the TAP-900//TAP-900 and TAP-1000//TAP-1000 symmetric devices (under CO_2). A 5-min voltage hold was applied to mitigate the influence of the relatively low electrical conductivity of TAP-900. Materials characterisation shows that TAP-900 contains a much higher nitrogen content than TAP-1000, along with a larger surface area and higher structural disorder. Correlated with these features, TAP-900 delivers slightly higher capacitances than TAP-1000 at low current densities (174 F g^{-1} for TAP-900 and 169 F g^{-1} for TAP-1000 both at 10 mA g^{-1}). However, its capacitance declines obviously as the current density increases, even with 5-min voltage holds. Similarly, the CO_2 adsorption capacity of TAP-900 peaks at a lower current density (*i.e.,* 30 mA g^{-1}) than TAP-1000 (*i.e.,* 100 mA g^{-1}), although its maximum uptake is marginally higher (188 $mmol_{CO2}$ kg^{-1} for TAP-900 at 30 mA g^{-1} and 177 $mmol_{CO2}$ kg^{-1} for TAP-1000 at 100 mA g^{-1}). These trends suggest that while increased nitrogen content, surface area, and disorder may enhance the thermodynamic capacities for charge storage and CO_2 capture, the limited electrical conductivity of TAP-900 strongly suppresses kinetic performance, particularly for SSA, which is actually a kinetic effect governed by competing CO_2 capture and release processes in symmetric supercapacitor cells. Here the electrical resistances of the TAP-900//TAP-900 symmetric cell and TAP-1000//TAP-1000 symmetric cell are around 160 and 17 Ω, respectively. These electrical

resistances were calculated from the IR_{drop} of their GCD curves at 30 mA g^{-1}, with all other cell components and cell configurations identical. This highlights the need for a trade-off: optimising carbon structure and heteroatom content while retaining sufficient electrical conductivity is essential for practical performance.

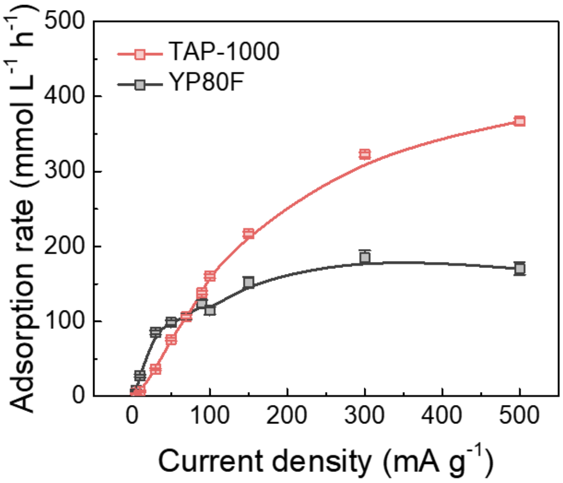

Figure S10. Characteristic dependence of volumetric CO_2 adsorption rate of the TAP-1000//TAP-1000 symmetric devices (using 1 M Na_2SO_4 (aq) as the electrolyte) and the YP80F//YP80F symmetric devices (using 1 M Na_2SO_4 (aq) as the electrolyte) (under CO_2, at the current densities from 5 to 500 mA g^{-1} in the negative charging mode, without voltage hold). Note: When normalised by the density of the as-prepared electrodes (YP80F electrodes: ~530 mg cm^{-3} and TAP-1000 electrodes: ~180 mg cm^{-3}), the corresponding maximum volumetric adsorption rates are 185 and 368 $mmol_{CO2}$ L^{-1} h^{-1}, respectively.

Table S1. Structure details of porous carbons.

	S_{BET}[a] ($m^2\ g^{-1}$)	V_t[b] ($cm^3\ g^{-1}$)	D_A[c] (nm)	I_D/I_G	C (at%) TPD	O (at%) TPD	H (at%) TPD	N (at%) TPD	Mg (wt%) ICP-MS
**TAP-900**	3293	2.70	2.90	1.03	86.4	2.2	3.4	8.0	0.12±0.02
**TAP-1000**	2830	2.73	3.13	1.17	94.0	1.6	2.0	2.4	0.24±0.05
**TAP-1100**	2618	2.12	2.89	1.18	94.3	1.6	2.0	2.1	0.42±0.08
**YP80F**	2324	1.14	1.13	1.16	94.1	1.1	4.8	N/A	N/A

Note: [a]Brunauer–Emmett–Teller (BET) specific surface area; [b]Total pore volume (NLDFT and slit pore model); [c]average pore diameter; I_D/I_G calculated from the intensity ratio of fitted D and G peaks; at% from TPD; wt% from ICP-MS.

Table S2. Detailed results of TPD measurements.

	H_2 (μmol/g)	CO (μmol/g)	CO_2 (μmol/g)	H_2O (μmol/g)	Total gas evolution (μmol/g) H_2+ H_2O + CO + CO_2
**TAP-900**	251.5	1675.6	248.8	637.6	2813.5
**TAP-1000**	316.8	966.5	209.4	324.2	1816.9
**TAP-1100**	428.9	989.6	194.6	238.4	1851.5
**YP80F**	1977.2	746.8	69.7	84.8	2878.5

	HCN (μmol/g)	N_2 (μmol/g)	NH_3 (μmol/g)	Total gas evolution (μmol/g) NH_3+ HCN + N_2
**TAP-900**	3877.5	2476.8	277.7	6632.0
**TAP-1000**	423.7	773.0	0.02	1197.0
**TAP-1100**	220.1	833.0	0.01	1053.0
**YP80F**	N/A	N/A	N/A	N/A

Table S3. Performance comparison between various CO_2 capture technologies.

	CO_2 adsorption capacity (mmol kg^{-1})	CO_2 adsorption rate (mmol kg^{-1} h^{-1})	CO_2 adsorption rate (mmol L^{-1} h^{-1})	Electrical energy consumption (kJ mol^{-1})
**SSA-1[8] (15% CO_2, 85% N_2)**	273	301	44	179
**SSA-2[9] (15% CO_2, 85% N_2)**	485	223	61	311
**Redox-active AzPy[10] (15% CO_2, 5% O_2, 80% N_2)**	N/A	N/A	N/A	120
**Redox-active quinone-1[11] (15% CO_2, 5% O_2, 80% N_2)**	N/A	N/A	N/A	50-200
**Redox-active quinone-2[12] (15-100% CO_2, 85-0% N_2)**	0.8-1.3 $\mu mol_{CO2}/\mu mol_{quinone}$	N/A	N/A	40–90
**BPMED[13] (pure CO_2)**	N/A	N/A	N/A	148−325
**EMAR[14] (15% CO_2, 85% N_2)**	0.12-0.62 mol_{CO2}/mol_{amine}	N/A	N/A	40-120
**PCET-1[15-16] (15% CO_2, 85% N_2)**	N/A	N/A	N/A	60-145
**PCET-2[17] (20% CO_2, 3-20% O_2, 77-60% N_2)**	N/A	N/A	860-1410	36–55
**Amine-based separation (flue gas)**	N/A	N/A	N/A	~65[13] ~90[18] ~120[19] ~100[20]
**This work – TAP-1000 (pure CO_2)**	~150	~2100	~370	~10
**This work – YP80F (pure CO_2)**	~66	~350	~185	~20

Notes: SSA (Supercapacitive swing adsorption), AzPy (4,4′-azopyridine), BPMED (Bipolar membrane electrodialysis), EMAR (Electrochemically mediated amine regeneration), PCET (Proton-coupled electron transfer with derivatized phenazine), N/A (not available). The volumetric capacity is normalized by the volume of the gas-exposed working electrode.

References

1. Gor, G. Y.; Thommes, M.; Cychosz, K. A.; Neimark, A. V., Quenched Solid Density Functional Theory Method for Characterization of Mesoporous Carbons by Nitrogen Adsorption. *Carbon* **2012**, *50*, 1583-1590.
2. Yoshii, T., et al., Quantitative and Qualitative Analysis of Nitrogen Species in Carbon at the Ppm Level. *Chem* **2024**, *10*, 2450-2463.
3. Binford, T. B.; Mapstone, G.; Temprano, I.; Forse, A. C., Enhancing the Capacity of Supercapacitive Swing Adsorption Co2 Capture by Tuning Charging Protocols. *Nanoscale* **2022**, *14*, 7980-7984.
4. Xu, Z.; Xie, F.; Wang, J.; Au, H.; Tebyetekerwa, M.; Guo, Z.; Yang, S.; Hu, Y. S.; Titirici, M. M., All-Cellulose-Based Quasi-Solid-State Sodium-Ion Hybrid Capacitors Enabled by Structural Hierarchy. *Adv. Funct. Mater.* **2019**, *29*, 1903895.
5. Zhu, S.; Li, J.; Toth, A.; Landskron, K., Relationships between Electrolyte Concentration and the Supercapacitive Swing Adsorption of Co2. *ACS Appl. Mater. Interfaces* **2019**, *11*, 21489-21495.
6. Zhu, S.; Li, J.; Toth, A.; Landskron, K., Relationships between the Elemental Composition of Electrolytes and the Supercapacitive Swing Adsorption of Co2. *ACS Appl. Energy Mater.* **2019**, *2*, 7449-7456.
7. Barrio, J., et al., Fenc Oxygen Reduction Electrocatalyst with High Utilization Penta-Coordinated Sites. *Adv. Mater.* **2023**, *35*, 2211022.
8. Bilal, M.; Li, J.; Landskron, K., Enhancing Supercapacitive Swing Adsorption of Co2 with Advanced Activated Carbon Electrodes. *Adv. Sustainable Syst.* **2023**, *7*, 2300250.
9. Bilal, M.; Li, J.; Guo, H.; Landskron, K., High-Voltage Supercapacitive Swing Adsorption of Carbon Dioxide. *Small* **2023**, *19*, 2207834.
10. Li, X.; Zhao, X.; Liu, Y.; Hatton, T. A.; Liu, Y., Redox-Tunable Lewis Bases for Electrochemical Carbon Dioxide Capture. *Nat. Energy* **2022**, *7*, 1065-1075.
11. Diederichsen, K. M.; Liu, Y.; Ozbek, N.; Seo, H.; Hatton, T. A., Toward Solvent-Free Continuous-Flow Electrochemically Mediated Carbon Capture with High-Concentration Liquid Quinone Chemistry. *Joule* **2022**, *6*, 221-239.
12. Voskian, S.; Hatton, T. A., Faradaic Electro-Swing Reactive Adsorption for Co2 Capture. *Energy Environ. Sci.* **2019**, *12*, 3530-3547.
13. Eisaman, M. D.; Alvarado, L.; Larner, D.; Wang, P.; Garg, B.; Littau, K. A., Co2 Separation Using Bipolar Membrane Electrodialysis. *Energy Environ. Sci.* **2011**, *4*, 1319-1328.
14. Wang, M.; Herzog, H. J.; Hatton, T. A., Co2 Capture Using Electrochemically Mediated Amine Regeneration. *Ind. Eng. Chem. Res.* **2020**, *59*, 7087-7096.
15. Jin, S.; Wu, M.; Gordon, R. G.; Aziz, M. J.; Kwabi, D. G., Ph Swing Cycle for Co2 Capture Electrochemically Driven through Proton-Coupled Electron Transfer. *Energy Environ. Sci.* **2020**, *13*, 3706-3722.
16. Jin, S.; Wu, M.; Jing, Y.; Gordon, R. G.; Aziz, M. J., Low Energy Carbon Capture Via Electrochemically Induced Ph Swing with Electrochemical Rebalancing. *Nat. Comm.* **2022**, *13*, 2140.
17. Pang, S.; Jin, S.; Yang, F.; Alberts, M.; Li, L.; Xi, D.; Gordon, R. G.; Wang, P.; Aziz, M. J.; Ji, Y., A Phenazine-Based High-Capacity and High-Stability Electrochemical Co2 Capture Cell with Coupled Electricity Storage. *Nat. Energy* **2023**, *8*, 1126-1136.
18. Hamdy, L. B.; Goel, C.; Rudd, J. A.; Barron, A. R.; Andreoli, E., The Application of Amine-Based Materials for Carbon Capture and Utilisation: An Overarching View. *Mater. Adv.* **2021**, *2*, 5843-5880.
19. Dutcher, B.; Fan, M.; Russell, A. G., Amine-Based Co2 Capture Technology Development from the Beginning of 2013—a Review. *ACS Appl. Mater. Interfaces* **2015**, *7*, 2137-2148.
20. Zito, A. M., et al., Electrochemical Carbon Dioxide Capture and Concentration. *Chem. Rev.* **2023**, *123*, 8069-8098.
